# Severe vitamin K deficiency-associated coagulopathy triggered by Clostridioides difficile infection and antibiotic-associated dysbiosis: A case report and literature review

**DOI:** 10.1007/s15010-026-02735-9

**Published:** 2026-01-21

**Authors:** Márk Kozák, Levente Majoros, Zoltán Panyiczki, Zsuzsa Bagoly, Rebeka Hodossy-Takács, Lili Virág Dobos, István Várkonyi

**Affiliations:** 1 Department of Infectology, Faculty of Medicine, University of Debrecen, 2-26 Bartok Bela Street, 4031 Debrecen, Hungary; 2 Division of Clinical Laboratory Sciences, Department of Laboratory Medicine, Faculty of Medicine, University of Debrecen, MTA-DE Lendület “Momentum” Hemostasis and Stroke Research Group, Debrecen, Hungary; 3 Physics Bachelor Student, École Polytechnique Fédérale de Lausanne (EPFL), Lausanne, Switzerland

**Keywords:** Vitamin K deficiency, Clostridioides difficile infection, Dysbiosis, Antibiotic-associated coagulopathy, Malnutrition

## Abstract

**Purpose:**

*Clostridioides difficile infection (CDI)* represents a major healthcare associated infection with potentially life-threatening complications. While gastrointestinal and systemic manifestations are well recognized, severe micronutrient deficiencies, particularly vitamin K deficiency are rarely described. We aimed to report a unique case of CDI-and antibiotic-associated dysbiosis and malabsorption leading to profound vitamin K deficiency and coagulopathy, thereby highlighting the clinical intersection between infection, microbiome disruption, and hemostasis.

**Methods:**

We report the clinical course, diagnostic work-up, and therapeutic management of an elderly female patient with CDI complicated by life-threatening coagulopathy. In addition, a narrative review of published case reports of antibiotic-associated vitamin K deficiency was performed to contextualize our findings.

**Results:**

The patient developed extensive subcutaneous hematomas with a severely deranged coagulation profile (PT > 100 s, INR > 8, markedly reduced activities of vitamin K–dependent factors). Normal liver function and preserved platelet count excluded disseminated intravascular coagulation and hepatic failure. The findings were consistent with severe vitamin K deficiency secondary to antibiotic-induced dysbiosis, malnutrition, and persistent diarrhea. High-dose intravenous vitamin K supplementation resulted in rapid normalization of coagulation parameters within 24 h, with subsequent clinical stabilization and resolution of bleeding manifestations.

**Conclusion:**

This case illustrates a rare but clinically significant complication of CDI: profound vitamin K deficiency–associated coagulopathy. Clinicians should maintain a high index of suspicion for vitamin K deficiency in elderly, malnourished, and antibiotic-exposed patients with CDI who present with unexplained coagulopathy or bleeding.

## Introduction

*Clostridioides difficile (C.difficile)* is a Gram-positive, spore-forming, obligate anaerobic bacterium that remains one of the most common causes of healthcare-associated infections worldwide. The clinical spectrum of *C. difficile* infection (CDI) ranges from asymptomatic carriage to life-threatening pseudomembranous colitis [1]. Major risk factors include advanced age, previous hospitalization, chronic kidney disease, use of proton pump inhibitors, and recent exposure to broad-spectrum antibiotics [2–5].

Beyond colitis, CDI may cause complications ranging from systemic inflammatory response to fulminant colitis, hypovolemia, toxic megacolon, and protein-losing enteropathy. [1, 6]. Severe gastroenteritis may lead to profound nutritional deficits and hypovitaminosis, with downstream effects on multiple organ systems [6, 7].

Vitamin K is essential for hemostasis as a cofactor in the γ-carboxylation of clotting factors II, VII, IX, and X as well as proteins C and S, and its deficiency may result in life-threatening bleeding and coagulopathy [8–11]. While phylloquinone (vitamin K1) is primarily derived from dietary sources such as green vegetables, menaquinones (vitamin K2) are produced by intestinal microbiota, predominantly in the ileum [12–16]. Antibiotic exposure, malabsorption syndromes, and disruption of gut microbial balance can markedly impair vitamin K synthesis and absorption, predisposing patients to deficiency [8].

Although micronutrient deficiencies are well documented in conditions such as inflammatory bowel disease (IBD) and chronic liver disease [17, 18], vitamin K deficiency associated with CDI is exceedingly rare. Only isolated case reports have described clinically significant coagulopathies triggered by the combined effects of antibiotic-induced dysbiosis and gastrointestinal malabsorption.

Here, we report the case of an elderly female patient who developed severe CDI complicated by antibiotic-associated dysbiosis, profound vitamin K deficiency, and life-threatening coagulopathy. This case highlights an under-recognized but clinically important intersection between infection, microbiome disruption, and micronutrient depletion.

The literature review was conducted as a narrative synthesis of current evidence on vitamin K deficiency–associated coagulopathy in the context of antibiotic therapy, gut dysbiosis, and Clostridioides difficile infection. A structured search was performed in PubMed/MEDLINE, Embase, Web of Science, and Google Scholar, using combinations of the following keywords: “vitamin K deficiency”, “hypovitaminosis K”, “PIVKA-II” “coagulopathy”, “bleeding”, “hemorrhage”, “hematoma” “antibiotic-associated”, “antibiotic-induced”, “broad-spectrum antibiotics” “dysbiosis”, “gut microbiota”, “intestinal flora” “Clostridioides difficile”, “Clostridium difficile”, “CDI” “malnutrition”, “protein-losing enteropathy”, “hypoalbuminemia”. The search was limited to English-language publications between January 2000 and July 2025, and included case reports, case series, narrative and systematic reviews, consensus statements, and observational studies. Reference lists of included articles were also screened to identify additional relevant reports.

## Case presentation

We report the case of an 81-year-old female nursing home resident with a history of dementia, atrial fibrillation, and Mobitz type II atrioventricular block. The patient was severely malnourished and cachectic due to advanced dementia (body mass index [BMI] 17.6 kg/m^2^; Mini Nutritional Assessment [MNA] score 2), was bedridden and unable to eat independently, and received assisted oral nutrition with high-energy, high-protein supplements, with daily nursing documentation of intake. She had previously received anticoagulation with the direct oral anticoagulant (DOAC) dabigatran, but was later transitioned to daily low-molecular-weight heparin (LMWH) prophylaxis. In April 2025, she was admitted to our department with urinary tract infection and right-sided pneumonia, for which she received intravenous ceftriaxone for seven days, resulting in complete clinical and microbiological resolution.

In May 2025, she was readmitted with severe hypotension and profuse watery diarrhea. Initial laboratory investigations demonstrated severe hypokalemia (2.3 mmol/L), acute kidney failure (creatinine 143 µmol/L, urea 23.5 mmol/L, eGFR 29 mL/min/1.73 m^2^), metabolic derangements including hypernatremia (148 mmol/L) and hypoalbuminemia (12.8 g/L). Leukocytosis (WBC 19.7 G/L, 88% neutrophils) and anemia (Hb 123 g/L) was observed. Inflammatory markers were significantly elevated **(**CRP 95 mg/L, procalcitonin 0.73 ng/mL). Stool antigen and toxin assays confirmed *C. difficile* infection, and oral vancomycin was initiated, followed by intravenous metronidazole due to ongoing diarrhea and rising inflammatory markers. She was managed on a general medical ward.

**Fig. 1:**
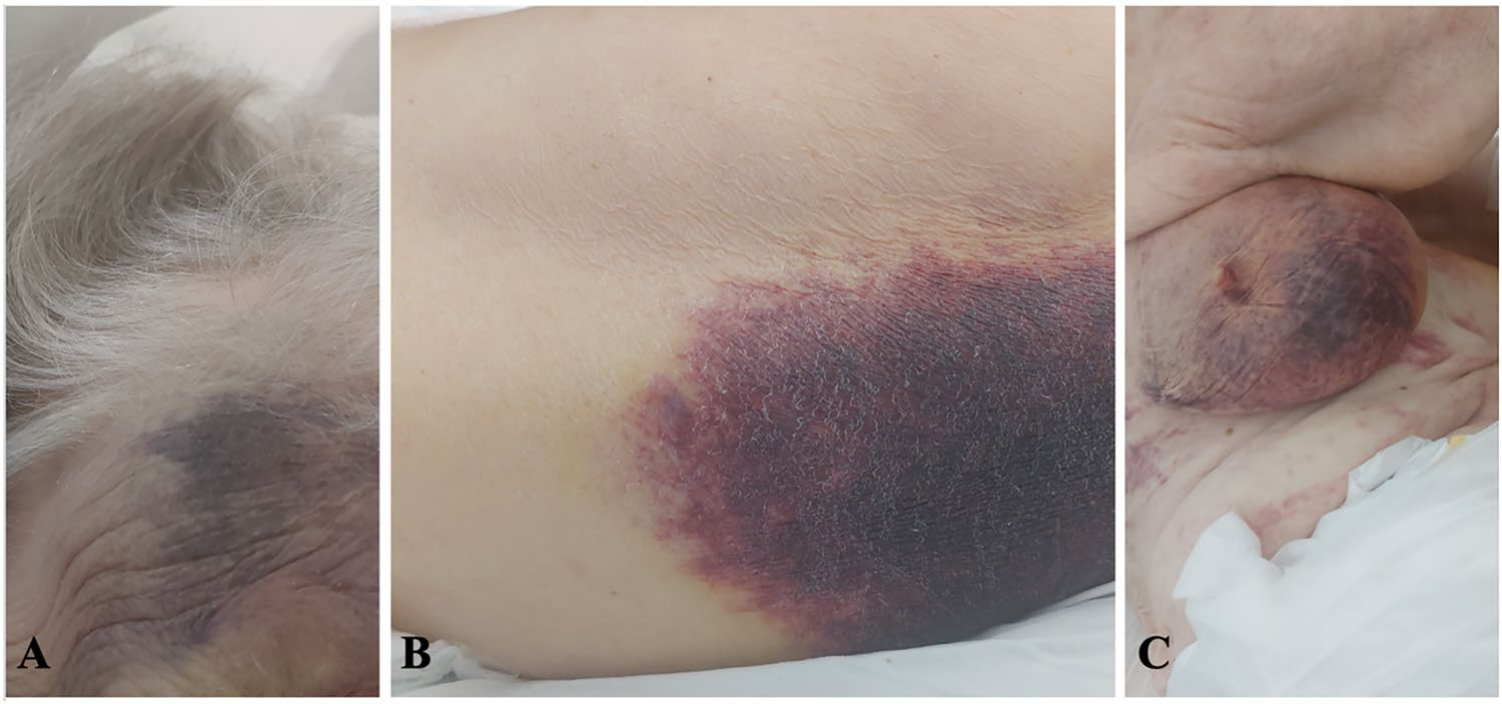
Rapidly developing hematomas in severe vitamin K deficiency–associated coagulopathy. Hematoma and diffuse ecchymosis on the forehead and scalp (**A**), confluent ecchymosis on the back with fascial extension (**B**), and hematoma of the right breast with marked swelling and discoloration (**C**)

During hospitalization, blood cultures grew methicillin-sensitive *Staphylococcus aureus* (MSSA) and methicillin-resistant *Staphylococcus aureus* (MRSA), prompting intravenous cefazolin therapy. Intermittently positive cultures for methicillin-resistant coagulase-negative staphylococci (MRCNS) were regarded as contaminants. Transthoracic echocardiography excluded infective endocarditis, and the bloodstream infection was considered healthcare-associated, most consistent with a peripheral intravenous catheter–related source. On day 20 of hospitalization, the patient developed rapidly progressive and extensive subcutaneous hematomas, involving the forehead and scalp, most prominently over the back with fascial plane extension, as well as the right breast with marked swelling (Fig. 1A–C). The administration of LMWH was discontinued. Surgical consultation recommended conservative management of the hematomas.

**Fig. 2:**
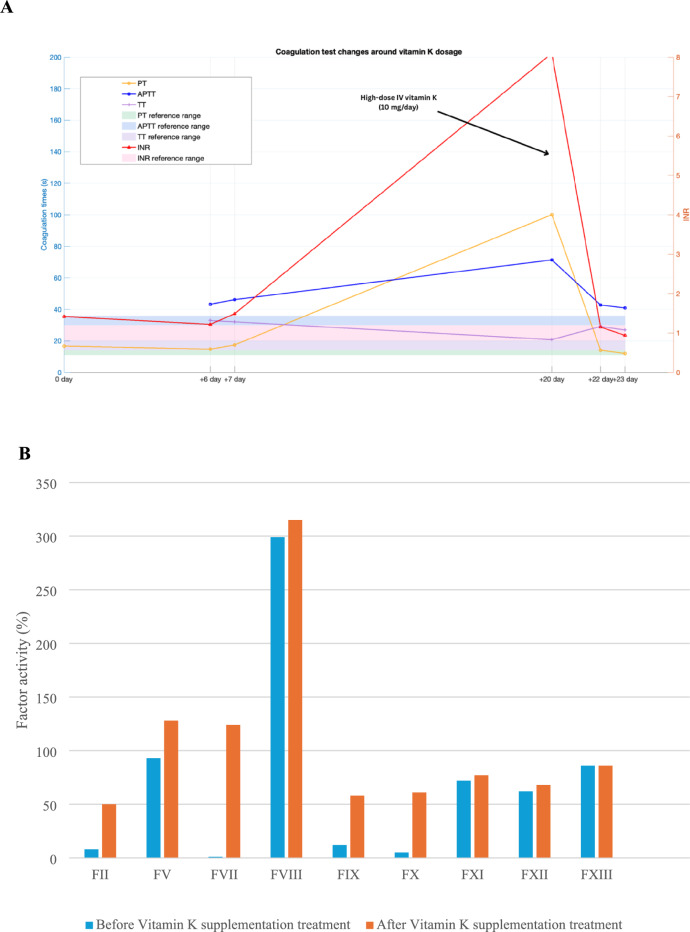
Screening tests of coagulation during the patient’s hospital stay (**A**). Coagulation factor activities before and after the administration of vitamin K therapy (**B**). **A** Orange line: prothrombin time, blue line: activated partial thromboplastin time, red line: International Normalized Ratio (INR), purple line: thrombin time. Blue zone: activated partial thromboplastin time reference range, pink zone: International Normalized Ratio (INR) reference range, purple zone: thrombin time reference range, green zone: prothrombin time reference range. Arrow: administration of high dose Vitamin K intravenously. **B** Blue box: factor activities before the administration of vitamin K, orange box: factor activities after the administration of vitamin K. *PT* prothrombin time, *INR* International Normalized Ratio, *APTT* activated partial thromboplastin time, *TT* thrombin time, *IV* intravenous

Coagulation screening tests revealed a profoundly disturbed hemostasis profile, with prolonged prothrombin time (PT) > 100 s (control: 9.5 s) (International Normalized Ratio [INR] > 8) and activated partial thromboplastin time (APTT) 73.2 s (control: 28.3 s). Thrombin time (TT) was not prolonged. Platelet count and fibrinogen level were preserved, and liver function tests remained within reference limits, excluding disseminated intravascular coagulation and hepatic failure. Mixing studies with healthy donors’ pooled plasma corrected both PT and APTT, effectively excluding inhibitors (Fig. 2A).

The activities of vitamin K–dependent clotting factors were markedly decreased (activity of factor II: 8%, factor VII: < 1%, factor IX: 12%, factor X: < 5%), while factors V and XIII activities were in the normal range (Fig. 2B).

Taken together, these findings were diagnostic of severe vitamin K deficiency–associated coagulopathy, most likely triggered by antibiotic-induced dysbiosis in the context of *Clostridioides difficile* colitis, malnutrition, and persistent diarrhea.

High-dose intravenous vitamin K (10 mg/day, Konakion) was administered, resulting in rapid normalization of PT, INR and APTT within 24 h. Subsequent coagulation assays demonstrated a substantial increase in vitamin K–dependent clotting factor activities (factors II, VII, IX, X), though levels remained below the reference range in some instances.

This partial biochemical recovery was sufficient to restore hemostatic balance, as reflected by stable coagulation values and the absence of further bleeding complications. Continued daily supplementation maintained stable coagulation values.

In parallel, intravenous albumin replacement and nutritional support were initiated orally to correct severe protein–calorie malnutrition and profound hypoalbuminemia. With resolution of diarrhea, normalization of inflammatory markers, recovery of renal function, and clearance of bloodstream infection, the patient’s hematomas regressed under conservative management, and she was discharged in stable condition.

## Discussion/mini review

### Pathophysiological links between CDI, dysbiosis, and vitamin K deficiency

*C. difficile* is a Gram-positive, spore-forming, obligate anaerobe causing a clinical spectrum from asymptomatic colonization to fulminant pseudomembranous colitis [1]. CDI is predominantly healthcare-associated, with established risk factors including recent hospitalization, advanced age, chronic kidney disease, proton pump inhibitor use, and prior exposure to broad-spectrum antibiotics particularly penicillins, cephalosporins, fluoroquinolones, and clindamycin although most antibiotic classes can predispose to infection by disrupting gut microbiota [2–5]. Transmission occurs mainly via the fecal–oral route and contaminated healthcare environments due to spore persistence, highlighting the importance of infection control and antibiotic stewardship [1].

C. difficile produces two major exotoxins (A and B), which disrupt the intestinal epithelium and induce neutrophil-driven inflammation, resulting in watery diarrhea. Hypervirulent strains (e.g., ribotype 027/NAP1/BI) produce higher toxin levels and an additional binary toxin, and are associated with increased severity, relapse rates, and fluoroquinolone resistance [1, 19].

Diagnosis relies on toxin-based assays within a two-step algorithm, with selective use of molecular testing, including polymerase chain reaction (PCR).Testing is indicated in patients with ≥ 3 unformed stools within 24 h and recent antibiotic exposure or healthcare contact, while imaging or endoscopy may aid in fulminant disease, and asymptomatic carriers should not be tested or treated [1, 19].

Clinical presentation varies with host immune status and strain virulence, ranging from watery diarrhea, abdominal pain, nausea, low-grade fever, and malabsorption to severe manifestations such as fulminant colitis, toxic megacolon, or protein-losing enteropathy. CT imaging may show colonic oedema and wall thickening, and severe CDI is defined by leukocytosis (> 15,000 cells/μL), hypoalbuminemia (< 3 g/dL), and a ≥ 1.5-fold rise in serum creatinine from baseline.

Management of CDI requires a stepwise therapeutic approach, including the discontinuation of non-essential antibiotics, targeted antimicrobial therapy, and, in selected cases, advanced interventions such as fecal microbiota transplantation or monoclonal antibody administration [1, 20, 21].

The intestinal tract plays a central role in nutrient digestion, absorption, detoxification, and immune regulation, processes influenced by diet, the intestinal microenvironment, and host physiological status. When mucosal integrity is compromised, as in IBD or acute gastroenteritis, patients become prone to dysbiosis, aberrant immune responses, and pathobiont colonization [22]. More than half of IBD patients exhibit micronutrient deficiencies, including vitamins D, B1, B6, B12, iron, and vitamin K. Accordingly, restoring gut microbiota–immune balance has emerged as a therapeutic target, with micronutrient supplementation demonstrating anti-inflammatory, antioxidant, and barrier-protective effects. [22].

Vitamin K is a fat-soluble vitamin with two biologically active forms: phylloquinone (K1), primarily derived from green vegetables, and menaquinones (K2), a heterogeneous group of compounds obtained from dietary sources such as eggs, cheese, and meat, but also synthesized by intestinal microbiota [15, 16]. Several bacterial species including *Bacteroides fragilis*, *Eggerthella lenta*, and *Lactococcus lactis* produce distinct menaquinones [12]. Dysbiosis reduces vitamin K–producing bacteria, thereby diminishing endogenous vitamin K2 synthesis, while the limited bioavailability of bacterial menaquinones further underscores the importance of adequate dietary intake[13, 14]. Vitamin K absorption depends on bile salts and pancreatic enzymes for micelle formation, followed by uptake in the small intestine [8]. Multiple conditions can impair this process, including intestinal injury, cholestatic liver disease, chronic kidney disease, and broad-spectrum antibiotic use [7, 8, 23, 24]. Antibiotic-induced disruption of gut microbiota markedly reduces endogenous vitamin K2 production. Prolonged broad-spectrum therapy can wipe out vitamin K–producing bacteria, predisposing to hypovitaminosis. Up to 25% of intensive care unit (ICU) patients on long-term antibiotics develop deficiency, and CDI itself often reflects profound dysbiosis, with mucosal injury and malabsorption further compounding vitamin K loss [12].

Vitamin K deficiency can cause clinically significant impaired γ-carboxylation of coagulation factors II, VII, IX, and X, as well as proteins C and S, may result in life-threatening bleeding [9–11].

Beyond depletion of gut flora, certain antibiotics directly impair vitamin K function. Cephalosporins containing N-methyl-thiotetrazole (NMTT) or 2-methyl-1,3,4-thiadiazole (MTD) side chains (e.g., cefoperazone, cefotetan, cefazolin) inhibit γ-carboxylation of vitamin K–dependent clotting factors, mimicking warfarin’s effect even in patients with adequate vitamin K stores [25–27].

Beyond hemostasis, vitamin K exerts anti-inflammatory effects through suppression of pro-inflammatory cytokines (e.g., interleukin-6 and tumor necrosis factor-α) [28, 29], reduces oxidative stress [30–32], and contributes to the regulation of the PC/PS pathway, thereby influencing not only coagulation, but also immune responses [33–38]. Deficiency of anticoagulant proteins has been associated with hypercoagulability and thromboembolic complications in Crohn’s disease [37, 38]. Thus, vitamin K plays multifaceted roles in vascular, hepatic, renal, and intestinal health, underscoring its importance as a micronutrient with both hemostatic and immunoregulatory functions [8, 38].

### Clinical and diagnostic implications

Beyond gastrointestinal manifestations, CDI may aggravate malnutrition and gut dysbiosis, leading to metabolic complications such as vitamin K deficiency. In severe cases, protein-losing enteropathy can further deplete albumin and coagulation factors, increasing coagulopathy risk, particularly in frail elderly patients. Although CDI-related hemostatic abnormalities are usually prothrombotic, rare hypocoagulable states have also been reported. [39]. Matthaiou et al. described an ICU case where broad-spectrum antibiotics and CDI together precipitated severe vitamin K deficiency coagulopathy (INR ~ 8) without other identifiable causes, illustrating how these factors can synergistically create a “perfect storm” for vitamin K–dependent dysfunction [12].

A growing number of case reports highlight that antibiotic-associated vitamin K deficiency–related coagulopathy is a multifactorial and clinically heterogeneous phenomenon. Reported cases involve elderly, malnourished, psychiatric, pediatric, and burn patients, with triggers ranging from cephalosporins with MTD/NMTT side chains**,** to rifampicin, carbapenems and prolonged broad-spectrum regimens and CDI infection. Clinical presentations vary from asymptomatic INR prolongation to life-threatening hemorrhage [12, 40–45], as summarized in Table 1.

**Table 1:** Reported case studies of antibiotic-associated vitamin K deficiency

Author (Year)	Patient	Suspected Trigger(s)	Presentation	Treatment	Outcome
Itagaki & Hagino (2019)	83-year-old female, cholangitis with sepsis, hypoalbuminemia (Japan)	Ampicillin/ sulbactam; restricted fat intake → vitamin K deficiency	Massive hemothorax after thoracentesis; PT 68–100 s, INR ↑, APTT immeasurable; platelets normal; PIVKA-II ~ 23,000 mAU/mL	IV vitamin K; RBC + FFP transfusions; coil embolization (7th–9th intercostal arteries)	Coagulation normalized; survived hemorrhagic shock; discharged in stable condition
Wu et al. (2022)	71-year-old male with pneumonia, frailty, multimorbidity (China)	Prolonged cephalosporins; malnutrition; mild liver dysfunction	Severe coagulopathy without bleeding; PT 136 s, INR > 10, APTT 54.8 s; platelets normal	IV vitamin K₁ 20 mg	Coagulation normalized within 24 h; no hemorrhage; discharged after infection resolution
Torres-Fernández et al. (2020)	8-month-old male infant, pulmonary TB (Spain)	Rifampicin-induced inhibition of vitamin K cycle	Multiple ecchymoses; PT 175 s, INR 15.6, APTT 160 s; FII 3%, FVII 2%, FIX 3%, FX 1%	IV vitamin K; FFP transfusion; oral vitamin K continued during TB therapy	Rapid normalization of coagulation; ecchymoses resolved; TB therapy completed successfully
Tatsumura et al. (2024)	70-year-old male with diabetes, postoperative spinal infection (Japan)	Malnutrition + prolonged cefazolin (MTD group), rifampicin, levofloxacin, TMP-SMX	Severe coagulopathy without bleeding; PT 74 s, INR 6.7, APTT 138 s; PIVKA-II 34,400 mAU/mL	IV vitamin K₁ 40 mg (× 4 days); nutritional optimization;	Coagulation normalized within 5 days
Matthaiou et al. (2023)	45-year-old male with aspiration pneumonia, catatonia, malnutrition (Greece)	Broad-spectrum antibiotics (piperacillin/tazobactam, meropenem, vancomycin, colistin, tigecycline, ampicillin/sulbactam) + vitamin E + CDI + malnutrition	Progressive coagulopathy without bleeding; PT 91.6 s, INR 7.9, APTT 66.2 s; FVII 8.4%	IV vitamin K₁ 10 mg; 2 units FFP; withdrawal of vitamin E	INR/PT normalized within 24 h; no hemorrhage; CDI treated; discharged after recovery
Nomoto et al. (2011)	62-year-old female with schizophrenia, catatonia, poor intake (Japan)	Prolonged antibiotics (ampicillin/sulbactam, cefmetazole); markedly reduced food intake	Severe asymptomatic coagulopathy; INR 7.08; PIVKA-II 83,750 mAU/mL; platelets normal	IV/PO vitamin K 20 mg/day;	INR normalized within 2 days; PIVKA-II decreased to 428 at 4 weeks; no bleeding
Liu et al. (2019)	40-year-old male with 45% TBSA burns + inhalation injury (China)	Prolonged meropenem (± tigecycline); suppression of vitamin K–producing flora	Unexpected bleeding (donor site, line removal); PT 15.6 s, INR 1.32, APTT 61.5 s; FII 39%, FVII 35%, FIX 45%, FX 28%	IV vitamin K 10–20 mg/day; daily FFP; prothrombin complex; withdrawal of meropenem	Coagulation normalized by day 39; multiple graft surgeries uneventful; discharged day 67

*APTT* Activated partial thromboplastin time, *CDI* Clostridioides difficile infection, *ECT* Electroconvulsive therapy, *FFP* Fresh frozen plasma, *INR*: International Normalized Ratio, *IV* Intravenous, *MTD*: 2-Methyl-1,3,4-thiadiazole side chain, *NMTT* N-methyl-thiotetrazole side chain, *PBD* Post-burn day, *PIVKA-II* Protein induced by Vitamin K absence or antagonist-II, *PT* Prothrombin time, *RBC* Red blood cell (transfusion), *TBSA* Total body surface area, TMP-*SMX* Trimethoprim–Sulfamethoxazole, *VKD* Vitamin K deficiency

Vitamin K deficiency–associated coagulopathy is characterized by a distinct laboratory pattern. The earliest and most sensitive finding is prolonged PT and INR, reflecting the short half-life of factor VII, while activated partial thromboplastin time APTT may remain normal or only mildly prolonged until factors II, IX, and X are significantly reduced [46]. Platelet counts and fibrinogen levels are typically preserved, distinguishing this condition from disseminated intravascular coagulation [47].

Diagnostic confirmation is supported by rapid normalization of coagulation parameters after parenteral vitamin K administration, favoring vitamin K deficiency over liver disease or coagulation inhibitors. Functional deficiency is further indicated by elevated protein induced by vitamin K absence or antagonist-II (PIVKA-II/des-γ-carboxy prothrombin, DCP), while a low factor II activity ratio measured by PT-based versus ecarin assays (FII/FIIE < 0.86) reflects impaired γ-carboxylation [48].

Platelet count and fibrinogen levels are typically preserved; proteins C and S may be reduced. D-dimer levels are usually within the normal range but may become elevated in the presence of large hematomas or concomitant systemic illness. Collectively, prolonged INR with preserved platelets and fibrinogen, selective depression of vitamin K–dependent factors, elevated PIVKA-II/DCP, and prompt correction after vitamin K therapy are diagnostic of vitamin K deficiency–associated coagulopathy [48].

Key laboratory abnormalities are summarized in Table 2.

**Table 2:** Main laboratory findings in vitamin K deficiency

Test	Typical findings
INR/PT	Prolonged (earliest and most sensitive marker, due to rapid fall of factor VII)
APTT	Normal or mildly prolonged; markedly prolonged in severe deficiency (low factor II, IX, X)
Fibrinogen	Normal (or increased); abnormal only if coexisting liver disease/consumptive state
Platelets	Normal (distinguishes VKD from DIC)
Vitamin K–dependent factors (II, VII, IX, X)	Reduced activity, other factors (e.g.: factor V, VIII, XI, XII) typically preserved
Anticoagulant proteins (PC, PS)	Reduced (rarely measured in bleeding patients in the clinical practice)
PIVKA-II (DCP)	Elevated; sensitive marker of VK deficiency; normalizes within days after supplementation
FII vs. FIIE ratio	Low FII/FIIE (< 0.86) or absolute difference ≥ 0.045 U/mL suggests VK deficiency; FIIE < 0.5 U/mL indicates liver disease rather than isolated VKD
D-dimer	Usually normal; may rise with large hematomas or concurrent illness

*INR* International Normalized Ratio, *PT* Prothrombin time, *APTT*: Activated partial thromboplastin time, *VKD* Vitamin K Deficiency, *DIC* Disseminated intravascular coagulation, *FIIE* Factor II activity measured with Ecarin reagent; *PIVKA-II/DCP* Protein induced by Vitamin K absence or antagonist-II (des-γ-carboxy prothrombin)

### Therapeutic considerations and preventive strategies

The cornerstone of management is vitamin K supplementation**,** with the route and dose tailored to severity. In mild to moderate cases without major bleeding, 1–10 mg vitamin K can be administered orally, subcutaneously, or intravenously (IV). In more severe coagulopathies, higher intravenous doses should be considered. If correction of the INR is incomplete, a repeat dose after 12 h is appropriate. In the presence of severe bleeding (e.g., intracranial hemorrhage on vitamin K antagonist therapy**)**, the recommended regimen is 10 mg vitamin K IV (infused ≤ 1 mg/min) combined with 50 units/kg prothrombin complex concentrate (PCC) and/or 1.2–4.8 mg recombinant activated factor VII (rFVIIa) IV [48].

A critical question is whether to discontinue or change the inciting antibiotic. In scenarios where the antibiotic is essential (e.g. to treat a serious infection), clinicians often continued therapy but with added vitamin K and close monitoring. For example, Tatsumura’s patient remained on cefazolin for weeks after correcting the coagulopathy, but with improved nutrition and no recurrence of coagulopathy [43]. In contrast, when a safer alternative is available, switching or discontinuation may lead to rapid recovery. This is particularly relevant for cephalosporins with NMTT or MTD side chains, such as cefotetan or cefoperazone, which directly impair vitamin K metabolism. In one report, cefoperazone-induced coagulopathy resolved promptly after the drug was withdrawn and vitamin K was administered [49].

The potential role of prophylactic vitamin K supplementation during antibiotic therapy remains debated. Pediatric studies have shown that prophylactic vitamin K was largely ineffective in preventing antibiotic-associated hypoprothrombinemia in critically ill children [50, 51]. However, some observational data suggest potential benefit in selected high-risk groups, such as preterm infants or exclusively breastfed neonates receiving prolonged antibiotic therapy [52]. Some studies in adults have suggested that supplementation can attenuate INR elevation in patients receiving cefoperazone/sulbactam [53–55] while other investigations failed to show a protective effect [56].

Therefore, no universal recommendation can be made. However, according to the proposal by Matthaiou et al., careful monitoring of coagulation parameters (INR, PT, and APTT) is strongly advised in patients receiving broad-spectrum antibiotics, especially those with malnutrition or malabsorption. They further suggest avoiding antibiotics with NMTT or MTD side groups in such patients whenever possible, and limiting vitamin E supplementation to cases with absolute indication, given its potential to interfere with vitamin K metabolism [12].

In the context of our case, optimal management of CDI was equally important in preventing recurrence and stabilizing the patient’s overall condition. Current guidelines recommend discontinuation of non-essential antibiotics and targeted therapy with oral vancomycin or fidaxomicin, with intravenous metronidazole reserved for severe or fulminant presentations [57]. In recurrent or refractory disease, strategies such as bezlotoxumab or faecal microbiota transplantation may be considered [20, 21].

Malnutrition and cachexia are common in frail patients with CDI, and nutritional support is essential to recovery. Early enteral feeding is preferred, as it maintains mucosal integrity, supports microbiota balance, and reduces complications [58]. Ongoing diarrhea and poor intake exacerbate deficiencies, particularly of fat-soluble vitamins. Clinical nutrition guidelines for long-term care settings emphasize a structured, individualized pathway: assessing hydration and electrolyte status, tailoring caloric intake to needs, and integrating micronutrient supplementation when deficiencies are suspected. Hydration can be delivered orally, enterally, or parenterally depending on clinical status, but restoration of enteral feeding is preferred whenever feasible to facilitate intestinal recovery [59].

## Conclusion

To our knowledge, this is the first detailed case report in which severe malnutrition, cephalosporin exposure, and active *Clostridioides difficile* infection coexisted and culminated in vitamin K deficiency–associated coagulopathy, supported by extensive hemostatic evaluation. This represents a rare but potentially fatal constellation, with only one comparable case previously reported [12]. The characteristic diagnostic constellation prolonged PT/INR and APTT, selective depletion of vitamin K–dependent factors, preserved fibrinogen and factor V, and rapid normalization after parenteral vitamin K was pivotal in distinguishing this condition from disseminated intravascular coagulation or hepatic failure.

From a clinical perspective, this report emphasizes the need for vigilance in frail, malnourished patients with CDI who receive broad-spectrum or high-risk antibiotics. Routine monitoring of coagulation parameters, early recognition of unexplained INR prolongation, and prompt vitamin K supplementation should be integrated into clinical practice. Nutritional support must be considered an essential part of management, not an adjunct.

Further research should aim to clarify the incidence and risk factors of vitamin K deficiency in CDI, to establish screening strategies for high-risk populations, and to evaluate whether prophylactic vitamin K supplementation or microbiome-directed therapies could reduce morbidity. Incorporating such preventive and diagnostic measures into clinical guidelines could help avert life-threatening complications in this vulnerable patient group.

### Limitations

This study has several limitations. First, it describes a single patient case, which restricts the generalizability of the findings. Second, although factor assays clearly demonstrated selective deficiency of vitamin K–dependent clotting factors and the rapid correction of coagulation abnormalities with vitamin K strongly supported the diagnosis, confirmatory biomarkers such as PIVKA-II were not available. Third, the literature review component was conducted as a narrative synthesis informed by a structured database search, but it was not a formal systematic review with predefined inclusion criteria or quantitative analysis. Therefore, the possibility of publication bias cannot be excluded, and further systematic studies are required to clarify the incidence, risk factors, and preventive strategies of vitamin K deficiency in patients with Clostridioides difficile infection.

## Data Availability

The authors will provide the raw data underlying the findings of this study upon reasonable request. For additional information, please contact the corresponding author.

## List of abbreviations

APTT: Activated partial thromboplastin time
BMI: Body Mass Index
CDI: *Clostridioides difficile* Infection
CT: Computer tomography
DCP: Des-γ-carboxy prothrombin
DOAC: Direct oral anticoagulant
FIIE: Factor II activity measured with Ecarin reagent
IBD: Inflammatory bowel disease
ICU: Intensive care unit
INR: International Normalized Ratio
IV: Intravenous
LMWH: Low-molecular-weight heparin
MNA: Mini Nutritional Assessment
MRCNS: Methicillin-resistant coagulase-negative staphylococcus
MRSA: Methicillin-resistant *Staphylococcus aureus*
MSSA: Methicillin-sensitive *Staphylococcus aureus*
MTD: 2-Methyl-1,3,4-thiadiazole
NMTT: N-methyl-thiotetrazole
PC: Protein C
PCC: Prothrombin complex concentrate
PCR: Polymerase chain reaction
PIVKA-II: Protein induced by vitamin K absence or antagonist-II
PS: Protein S
PT: Prothrombin time
rFVIIa: Recombinant activated factor VII
TT: Thrombin time
